# Clinical and genetic spectrum of pediatric mitochondrial disorders in China: insights from a 47-case genetically confirmed cohort

**DOI:** 10.1186/s13023-025-04180-7

**Published:** 2026-01-08

**Authors:** Fan Yang, Ruen Yao, Guoying Chang, Jiayue Hu, Biyun Feng, Libo Wang, Feihan Hu, Yiguo Huang, Shuo Wu, Tingting Yu, Yu Ding, Xiumin Wang

**Affiliations:** 1 Department of Clinical Research Ward, Shanghai Children’s Medical Center, Shanghai Jiao Tong University School of Medicine, Shanghai, 200127 China; 2 Department of Endocrinology, Genetics and Metabolism, Shanghai Children’s Medical Center, Shanghai Jiao Tong University School of Medicine, Shanghai, 200127 China; 3 Department of Medical Genetics and Molecular Diagnostics Laboratory, Shanghai Children’s Medical Center, Shanghai Jiao Tong University School of Medicine, Shanghai, 200127 China

**Keywords:** Mitochondrial disorders, Pediatric, Genotype–phenotype correlation, Heteroplasmy, Neuroimaging

## Abstract

**Background:**

Primary mitochondrial disorders (MDs) are genetically and clinically heterogeneous metabolic diseases caused by mitochondrial DNA (mtDNA) or nuclear DNA (nDNA) mutations. Pediatric MDs pose diagnostic challenges due to variable onset, multisystem involvement, and complex genotype–phenotype correlations.

**Methods:**

We retrospectively analyzed 69 children with suspected MDs at Shanghai Children’s Medical Center (2015–2025). Genetic diagnoses were established through targeted next-generation sequencing (NGS) combined with clinical, biochemical, and neuroimaging evaluation. Forty-seven patients were genetically confirmed and classified into mtDNA and nDNA groups for comparison.

**Results:**

The diagnostic yield was 68% (47/69). mtDNA mutations accounted for 64% (30/47), with *MT-TL1* m.3243 A > G being the most frequent (60%), followed by *MT-ATP6* m.8993T > C and *MT-ND6* m.14484T > C. nDNA mutations (36%) affected 12 genes, mostly related to complex I. Common phenotypes included MELAS (45%) and Leigh syndrome (LS) (28%). Compared to nDNA cases, mtDNA patients had higher epilepsy (64.5% vs. 43.8%) and stroke-like episodes (58.1% vs. 31.2%), while nDNA cases had earlier onset (1.15 vs. 5.42 years, *P* < 0.001) and more hypoglycemia. *MT-TL1* pedigrees showed maternal inheritance, phenotypic variability, and generational worsening. Mutation load was higher in *MT-ATP6*-related LS than in *MT-TL1*-related MELAS (*P* = 0.008). Serial neuroimaging revealed syndrome-specific progression. All six fatal cases exhibited a “neuroimaging–metabolic–cardiac/respiratory failure” triad.

**Conclusions:**

Chinese pediatric MDs show marked genetic and phenotypic heterogeneity. Recognizing genotype–phenotype patterns, longitudinal imaging, and critical risk triads may improve diagnosis, management, and patient selection for future trials.

## Introduction

 Primary mitochondrial disorders (MDs) are rare, genetically determined metabolic diseases caused by pathogenic variants in either mitochondrial DNA (mtDNA) or nuclear DNA (nDNA), resulting in impaired oxidative phosphorylation (OXPHOS) and cellular energy deficiency [1, 2]. Owing to the ubiquitous role of mitochondria in energy metabolism, these disorders can affect virtually any organ system, with the central nervous system, skeletal muscles, heart, liver, endocrine glands, and gastrointestinal tract most frequently involved [3]. The clinical spectrum is highly heterogeneous, ranging from isolated single-organ involvement to complex multisystem syndromes, and disease onset can occur at any age.

Epidemiological studies report a prevalence of 1 in 4,300 to 1 in 10,000 in Western countries, while data from Hong Kong indicate an incidence of 1.02 per 100,000 and a prevalence before the age of 18 of 5.24 per 100,000 [4, 5]. Such findings highlight notable geographical variations in disease burden. However, due to the complex and variable presentation of MDs, limited clinician awareness, and inconsistent access to advanced genetic testing, many patients face diagnostic delays or misdiagnosis, which can significantly affect prognosis and quality of life. Although certain subtypes, such as coenzyme Q biosynthesis defects, may benefit from targeted supplementation, most patients rely solely on supportive measures, as no curative therapy currently exists [6, 7]. The development of effective interventions is hindered by the diversity of over 300 reported causative genes and the incomplete understanding of pathogenic mechanisms [8].

In recent years, next-generation sequencing (NGS) has greatly facilitated the genetic diagnosis of MDs in China [9, 10]; however, comprehensive analyses focusing on the clinical and genetic features of pediatric patients remain scarce [8, 11]. To address this gap, we conducted a decade-long, single-center retrospective study of children with genetically confirmed MDs at Shanghai Children’s Medical Center, aiming to characterize the mutation spectrum, delineate genotype–phenotype correlations, explore the relationship between heteroplasmy and clinical severity, and identify prognostic indicators through dynamic neuroimaging follow-up and detailed examination of fatal cases.

## Methods

### Study design and ethical approval

This retrospective study included pediatric patients evaluated for suspected MDs at Shanghai Children’s Medical Center between January 2015 and January 2025. The study was approved by the Ethics Committee of Shanghai Jiao Tong University School of Medicine (SCMCIRB-K2020060-1), and written informed consent was obtained from the legal guardians of all participants. Patients were identified based on clinical suspicion of MD, which was defined by the presence of multisystem involvement such as neurological, muscular, endocrine, gastrointestinal, or cardiac manifestations, in combination with persistent lactic acidosis, neuroimaging abnormalities, or a family history suggestive of mitochondrial or metabolic disorders.

### Clinical data collection

Comprehensive demographic, clinical, and laboratory data were extracted from electronic medical records, including age at onset, presenting symptoms, disease course, biochemical profiles, neuroimaging results, and findings from cardiac and gastrointestinal evaluations. Structured interviews with parents or caregivers were conducted by trained attending physicians using a standardized MD assessment protocol. The likelihood of MD was determined using the Mitochondrial Disease Criteria (MDC) scoring system proposed by Morava et al. [12], which incorporates clinical manifestations across organ systems, metabolic and neuroimaging findings, and histopathological evidence from muscle biopsy where available. Cases were categorized as unlikely (score 1), possible [2–4], probable [5–7], or definite [8–12].

### Genetic testing and interpretation

Genetic testing was performed for patients meeting at least one of the following: an MDC score of 5 or higher; clinical features involving two or more major systems; persistent elevation of plasma lactate above 5 mmol/L; unexplained seizures, stroke-like episodes, or neurodevelopmental regression; or a family history of mitochondrial or metabolic disease. mtDNA sequencing was prioritized in cases with classical mitochondrial phenotypes or maternal inheritance patterns, whereas whole-exome sequencing (WES) was applied in patients with atypical presentations, negative mtDNA results, or suspected nuclear gene involvement. Variants were classified according to the American College of Medical Genetics and Genomics (ACMG) guidelines, and only pathogenic or likely pathogenic variants were included in the final analysis. Heteroplasmy levels were quantified using NGS read depth analysis from peripheral blood samples.

### Phenotypic classification

Phenotypes were assigned according to internationally accepted criteria for MELAS, Leigh syndrome (LS), mitochondrial myopathy, complex I deficiency, Barth syndrome, *MPV17-*related hepatocerebral disease, *SLC25A26* deficiency, and unclassified forms.

### Statistical analysis

Statistical analyses were conducted using SPSS version 26.0. Continuous variables were expressed as mean ± standard deviation or median (range) depending on distribution, while categorical variables were presented as counts and percentages. Between-group comparisons were performed using Pearson’s chi-square or Fisher’s exact test for categorical variables and the Mann–Whitney U test for continuous variables. A two-tailed P-value below 0.05 was considered statistically significant.

## Results

### Diagnostic yield and mutation spectrum

**Fig. 1:**
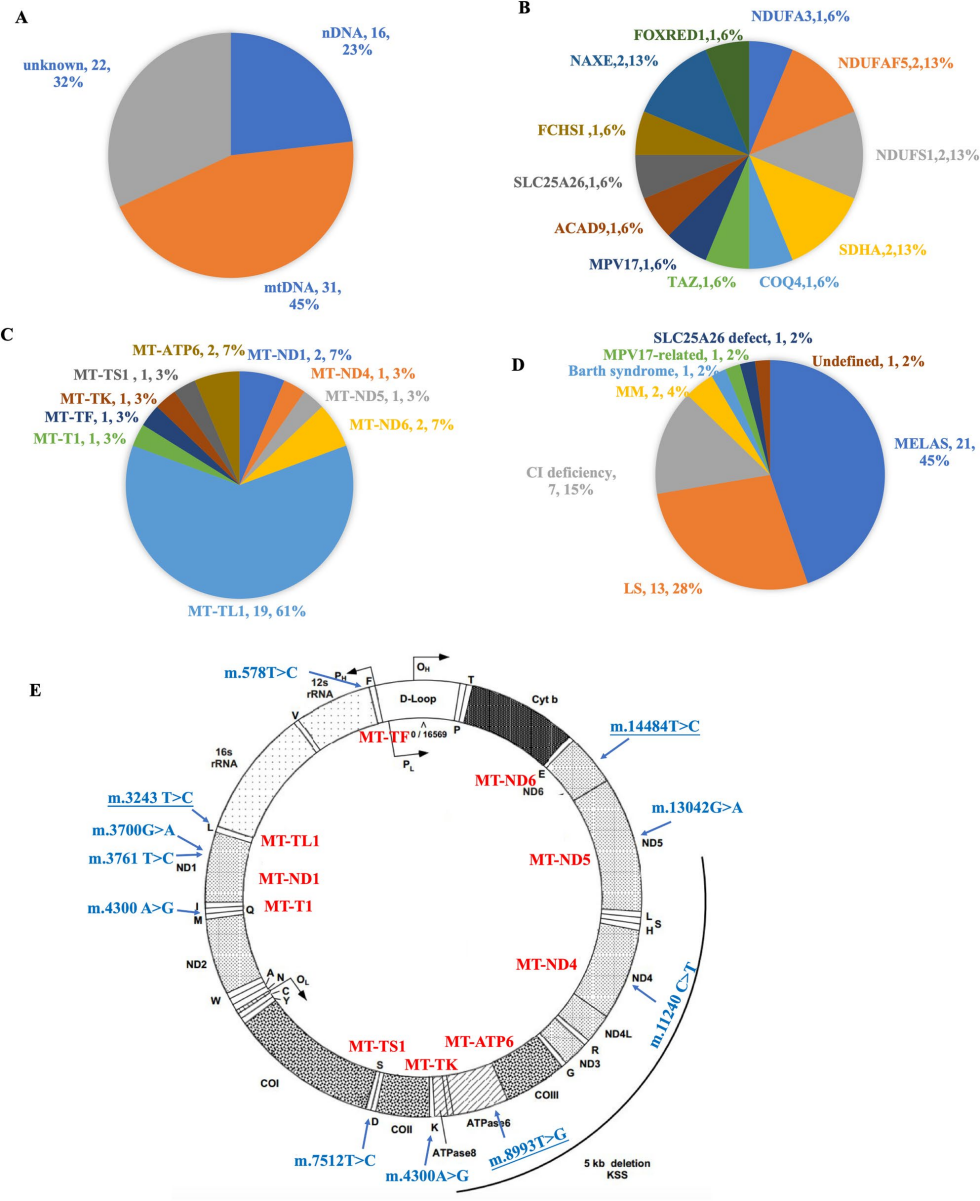
Genetic findings in 69 pediatric patients with suspected mitochondrial disorders. (**A**) Diagnostic yield: 47 of 69 patients (68%) had a confirmed genetic diagnosis, while 22 (32%) remained undiagnosed. (**B**) Spectrum of nuclear DNA (nDNA) mutations in 17 patients, with most involving complex I-related genes. (**C**) Spectrum of mitochondrial DNA (mtDNA) mutations in 30 patients, showing a predominance of the MT-TL1 m.3243 A > G variant (60%). (**D**) Distribution of clinical phenotypes in genetically confirmed cases (*n* = 47), with MELAS and LS as the most common subtypes.(**E**) Distribution of mtDNA variant types, including rare single-nucleotide variants. Variants in MT-ND1 (m.3700G > A and m.3761T > C) were each detected in single patients. The m.8993T > C (MT-ATP6) and m.14484T > C (MT-ND6) variants were identified in each of two patients

Among the 69 children with suspected MD, a genetic diagnosis was confirmed in 47 cases, yielding an overall diagnostic rate of 68% (Fig. 1A). Of these, 30 patients (64%) carried mtDNA mutations and 17 (36%) harbored nDNA mutations. 

In the mtDNA group, ten distinct pathogenic or likely pathogenic variants were detected (Fig. 1C). The MT-TL1 m.3243 A > G variant predominated, identified in 19 patients (60%), followed by MT-ATP6 m.8993T > C and MT-ND6 m.14484T > C (two cases each), with the remainder being rare single-nucleotide variants (Fig. 1E). In the nDNA group, 12 different genes were implicated, most commonly complex I-related genes such as NDUFS1, NDUFAF5, and SDHA, along with structural/metabolic genes such as *TAZ*, *MPV17*, and *SLC25A26* (Fig. 1B).

### Clinical phenotypes and age distribution

The cohort exhibited eight distinct phenotypes (Fig. 1D). MELAS was the most frequent (21 cases, 45%), including two with overlapping NARP features, followed by LS (13 cases, 28%). Mitochondrial myopathy and complex I deficiency each occurred in seven patients, while Barth syndrome, *MPV17*-related disease, *SLC25A26* deficiency, and mitochondrial cardiomyopathy were rare presentations.

**Fig. 2:**
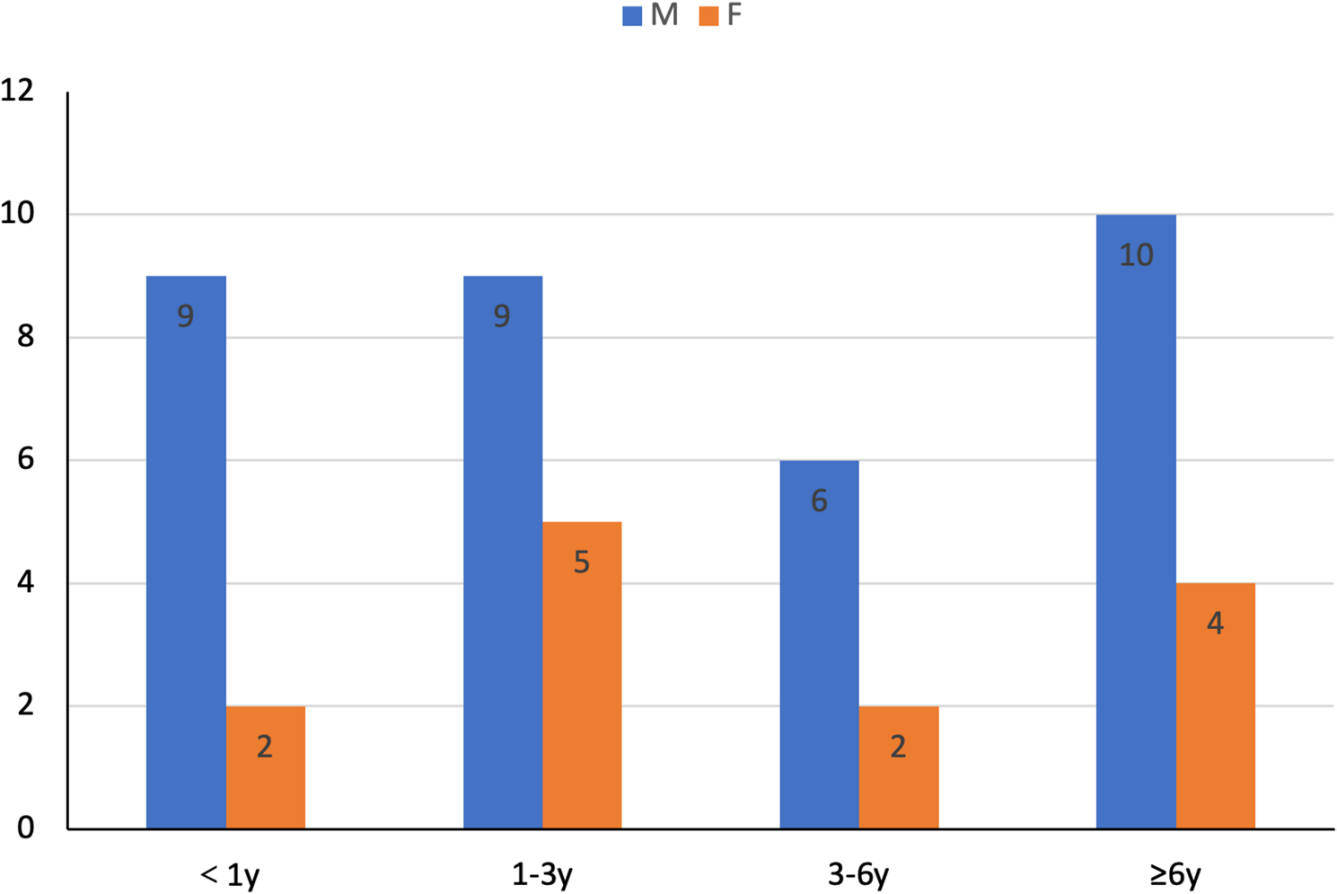
Age and sex distribution of 47 genetically confirmed pediatric mitochondrial disorder cases. Age at onset is categorized as < 1 year, 1–3 years, 3–6 years, and ≥ 6 years. Male predominance was observed overall (34 males, 13 females) and across all age groups

Age at onset ranged from the neonatal period to 13 years, showing a bimodal distribution: 26 patients (55.3%) presented before 3 years of age, including 12 (25.5%) within the first year, while 14 (29.8%) had onset at ≥ 6 years (Fig. 2). Males predominated overall (34:13), a pattern consistent across age groups.

### Comparison between mtDNA and nDNA groups

As summarized in Table 1, nDNA mutation carriers had a significantly earlier onset than mtDNA carriers (1.15 ± 1.46 years vs. 5.42 ± 4.37 years, *P* < 0.001). Epilepsy occurred more often in the mtDNA group (64.5% vs. 43.8%), as did stroke-like episodes (58.1% vs. 31.2%), although the latter did not reach statistical significance (*P* = 0.081). Hypoglycemia was observed exclusively in the nDNA group, whereas liver dysfunction, gastrointestinal symptoms, and cardiac abnormalities occurred with similar frequency in both groups.

**Table 1:** Comparison of demographic, clinical, and metabolic features between MtDNA and nDNA mutation groups in pediatric mitochondrial disorders (*n* = 47)

Variable	mtDNA (*n* = 31)	nDNA (*n* = 16)	*P*-value
Sex (male)	23 (74.2%)	11 (68.8%)	0.693
Family history	9 (22.6%)	3 (18.8%)	0.444
Age at onset (years)	5.42 ± 4.37	1.15 ± 1.46	**< 0.001**
Score	6.23 ± 1.06	6.43 ± 0.96	0.520
Lactate level (mmol/L)	4.76 ± 2.38	6.55 ± 3.17	0.058
Short stature	17 (54.8%)	7 (43.8%)	0.471
Epilepsy	20 (64.5%)	7 (43.8%)	0.172
Stroke-like episodes	18 (58.1%)	5 (31.2%)	0.081
Abnormal brain imaging	20/27 (74.1%)	11/11 (100.0%)	0.062
Developmental delay	17 (54.8%)	13 (81.2%)	0.074
Ocular problems	4 (12.9%)^**a**^	0 (0.0%)	0.133
Hearing impairment	4 (12.9%)	2 (12.5%)	0.969
Hypertrichosis	5 (16.1%)	0 (0.0%)	0.089
Elevated myocardial enzymes	8/25 (32.0%)	2/11 (18.2%)	0.392
Arrhythmia	5 (16.1%)	1 (6.2%)	0.332
Cardiac structural abnormalities	4/25 (16.0%)	3/11 (27.3%)	0.433
Heart failure	1 (3.2%)	2 (12.5%)	0.264
Abdominal pain/vomiting	24 (77.4%)	11 (68.8%)	0.518
Abnormal liver function	8 (25.8%)	5 (31.2%)	0.692
Abnormal blood glucose	0 (0.0%)	1 (6.2%)	0.159
Abnormal thyroid function	2 (6.5%)	3 (18.8%)	0.192

^a^There were 4 cases of ocular lesions. Among them, 2 cases were caused by optic nerve involvement, resulting in blurred vision or even blindness, and 2 cases were due to involvement of extraocular muscles, causing ptosis of the eyelids.

### Family history of diabetes and phenotypic heterogeneity in *MT-TL1* m.3243 A > G

**Fig. 3:**
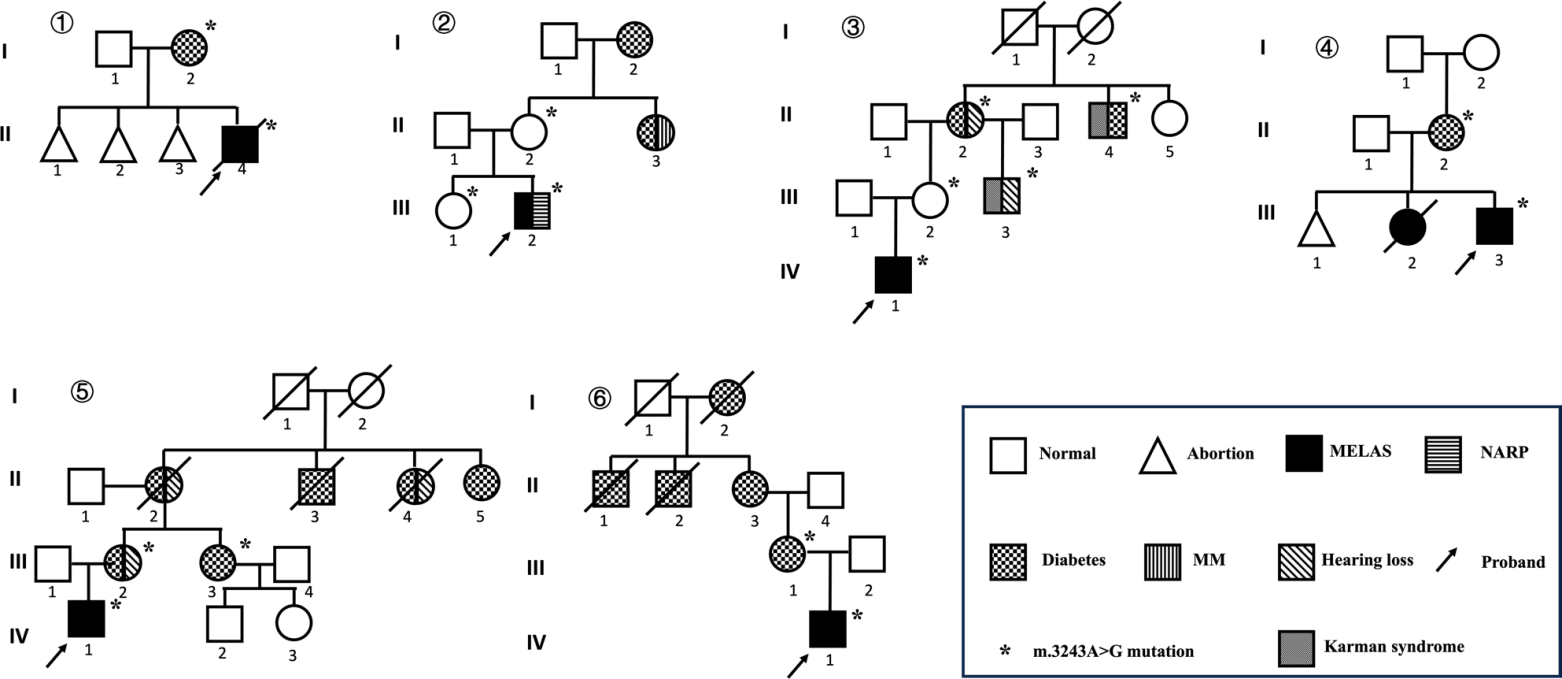
Pedigree analysis of six unrelated families with MT-TL1 m.3243 A > G mutation Pedigrees illustrate strict maternal inheritance, marked phenotypic heterogeneity ranging from isolated diabetes to multisystem MELAS/NARP, incomplete penetrance, and evidence of generational aggravation

Six unrelated families carrying the m.3243 A > G variant were analyzed, all of which had a documented family history of diabetes mellitus among maternal relatives. Notably, none of the probands in these families had developed diabetes at the time of last follow-up, despite exhibiting multisystem involvement and confirmed pathogenic variants. These pedigrees demonstrated strict maternal inheritance, pronounced phenotypic heterogeneity, incomplete penetrance, and evidence of generational aggravation. (Fig. 3).

### Mutation load and clinical correlations

In mtDNA mutation carriers (Table 2), heteroplasmy levels were not significantly associated with common symptoms such as epilepsy, stroke-like episodes, developmental delay, or short stature.

**Table 2:** Association between mitochondrial DNA mutation load and high-frequency clinical symptoms in pediatric patients with MtDNA mutations (*n* = 31). High-frequency symptoms are defined as those present in ≥ 10 cases

Symptom	Symptomatic Group Mean Mutation Load ± SD	Asymptomatic Group Mean Mutation Load ± SD	*p*-value
Epilepsy (*n* = 20)	0.74 ± 0.17	0.84 ± 0.19	0.130
Short stature (*n* = 17)	0.75 ± 0.18	0.81 ± 0.17	0.367
Stroke-like episodes (*n* = 18)	0.74 ± 0.17	0.83 ± 0.17	0.119
Neuroimaging abnormalities (*n* = 20)	0.76 ± 0.18	0.77 ± 0.19	0.905
Developmental delay (*n* = 17)	0.78 ± 0.18	0.76 ± 0.18	0.723
Abdominal pain/vomiting (*n* = 24)	0.77 ± 0.20	0.80 ± 0.70	0.678

### Longitudinal neuroimaging findings

**Fig. 4:**
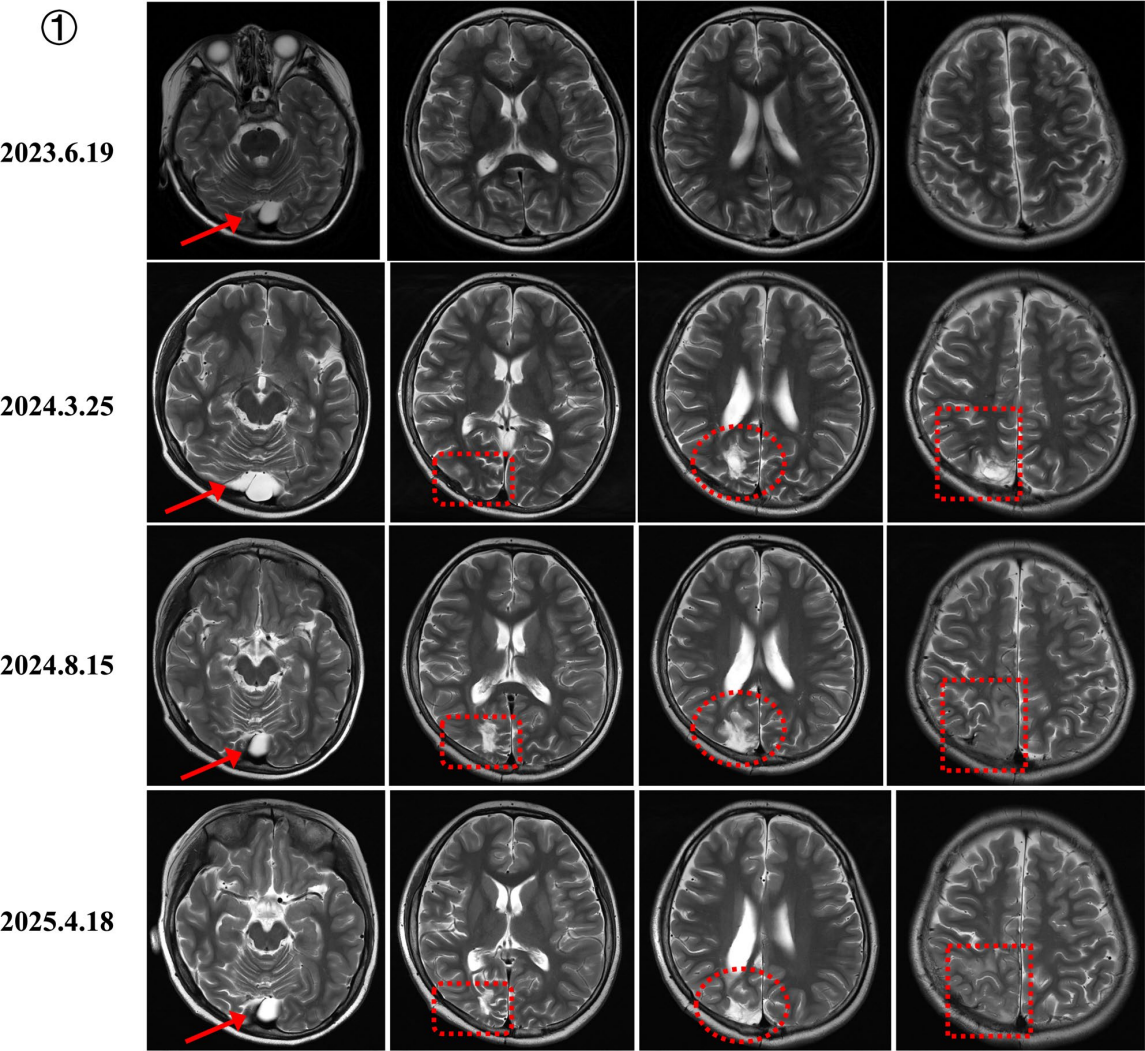
Serial brain MRI in a MELAS patient with MT-TL1 m.3243 A > G mutation (2023–2025). Progressive cortical involvement is shown, correlating with refractory seizures and motor decline. Improvement was temporarily achieved after SEEG-guided thermocoagulation (July 2023) and subtotal lobectomy (January 2024), followed by recurrence and further progression in the right hemisphere (April 2025)

**Fig. 5:**
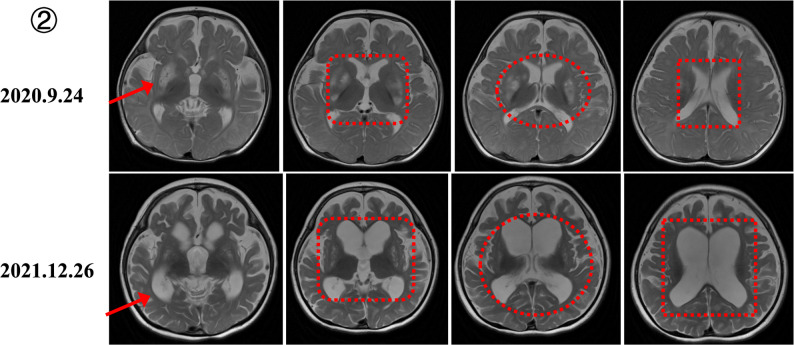
MRI progression in LS due to MT-ATP6 m.8993T > G mutation (2020–2021). Images demonstrate progressive ventricular enlargement, persistent basal ganglia lesions, and evolving cortical atrophy during 15 months of follow-up

**Fig. 6:**
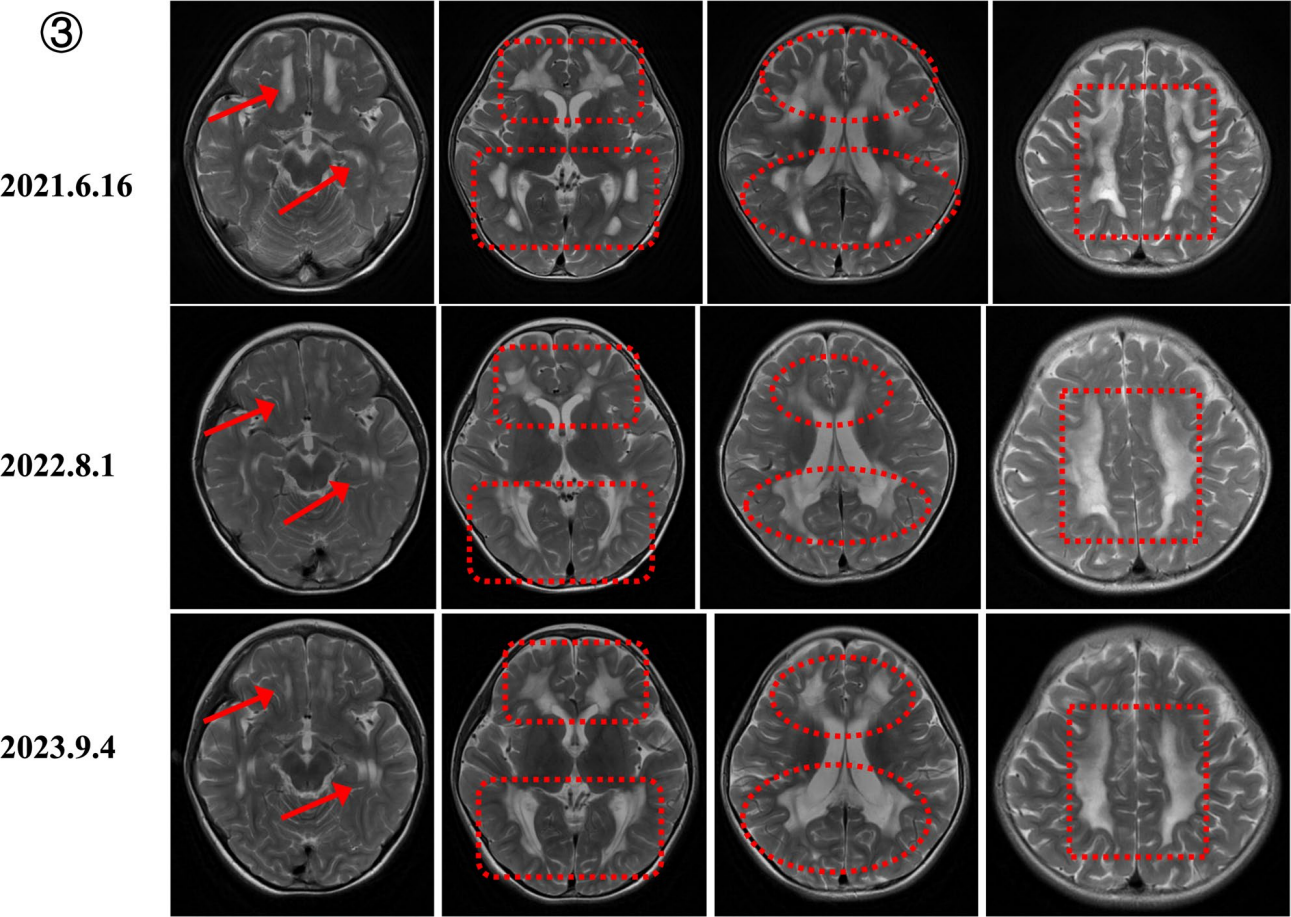
MRI evolution in NDUFS1-related complex I deficiency (2021–2023). (**A**) Initial cortical metabolic injury with cystic changes (2021). (**B**) Development of symmetric leukoencephalopathy involving the corpus callosum (2022). (**C**) Progressive white matter involvement (2023), reflecting a neurodegenerative trajectory refractory to mitochondrial therapy

Serial MRI in MELAS demonstrated stroke-like lesions extending beyond vascular territories, correlating with seizure exacerbations, and transient postoperative improvement after SEEG-guided thermocoagulation or lobectomy, followed by deterioration after infections (Fig. 4). *MT-ATP6*–related LS showed progressive ventricular enlargement, persistent basal ganglia lesions, and cortical atrophy (Fig. 5). *NDUFS1*-related complex I deficiency evolved from cortical metabolic injury with cystic changes to symmetric leukoencephalopathy involving the corpus callosum (Fig. 6).

### Fatal cases and prognostic indicators

Six patients died during follow-up (Table 3). Five of these six had required ventilatory support during the disease course, and five died from cardiac or circulatory failure. All fatal cases exhibited a consistent “neuroimaging–metabolic–cardiac/respiratory failure” triad, characterized by distinctive neuroimaging abnormalities, marked hyperlactatemia (≥ 6 mmol/L), and either significant cardiac involvement or terminal respiratory failure. This composite feature was strongly associated with adverse outcomes and may serve as a high-risk prognostic indicator.

**Table 3:** Clinical characteristics, neuroimaging findings, and causes of death in six fatal pediatric mitochondrial disorder cases. Includes both genetically confirmed and clinically diagnosed cases

Case	Genetic Diagnosis	Onset – Death	Key Clinical Manifestations	Imaging Features	Lactate (mmol/L)	Direct Cause of Death
1	NDUFS1 c.233G > A / c.616G > T	0 days– 12 days	Intrauterine growth restriction; preterm birth; hypertrophic cardiomyopathy; heart failure; vomiting	Head CT: Diffuse hypoxic–ischemic encephalopathy	12	Neonatal circulatory failure, Respiratory failure
2	NAXE c.310_314dup / c.713T > G	1 year – 4 years 3 months	Developmental regression with progressive cerebral dysfunction; hypothyroidism; central diabetes insipidus;	MRI: Periventricular white matter T2 hyperintensity	10.7	Respiratory failure
3	MT-TL1 m.3243 A > G	2 years 5 months – 4 years	Patent ductus arteriosus; antral gastritis; abnormal maternal abortion; seizures; hepatic dysfunction; ventricular tachycardia; short stature	MRI: Occipital gray–white matter abnormalities	6	Ventricular tachycardia, Respiratory failure
4	TAZ c.758G > A	2 years – 3 years	Developmental delay; recurrent fever; heart failure; myocardial injury; hepatomegaly; exercise intolerance	MRI: Periventricular punctate ischemic lesions	6.2	Refractory heart failure
5	Clinically diagnosed	1 year – 11 years	Neurodegeneration; decreased activity of respiratory chain complexes I and III; hyperlactatemia; severe pneumonia; gastrointestinal bleeding; hypertrophic cardiomyopathy	Serial MRI: Basal ganglia lesions → global cerebral atrophy	7	Respiratory failure
6	Clinically diagnosed	3 months – 3 months	Sibling neonatal sudden death; severe pneumonia; metabolic acidosis; cardiac insufficiency; myocardial injury; hyperlactatemia; cardiomyopathy	MRS: Lactate doublet peak at posterior horns	7.1	Circulatory collapse / respiratory failure

## Discussion

This 10-year, single-center retrospective analysis provides an in-depth characterization of the genetic and phenotypic spectrum of pediatric mitochondrial disorders (MDs) in China. Our results confirm the marked heterogeneity in both genotypes and clinical presentations while also identifying novel patterns of disease progression and high-risk prognostic indicators (Table 4 and Table 5). These findings expand the current knowledge of MDs in the Chinese pediatric population and provide a framework for genotype-informed clinical management.

**Table 4:** Clinical and genetic characteristics of pediatric patients with nuclear DNA (nDNA) mutations (*n* = 17)

ID	SEX	Age of onset	Clinical presentation	Score	Lactate (mmol/L)	Imaging Findings	Family history	Gene	Phenotype	Mutation and Heteroplasmy Level
1	M	Infancy	Developmental delay, exercise intolerance, short stature, vomiting/spitting up, positive pyramidal tract signs; sister has the same symptoms	6	U	U	Y	SDHA	LS	c.456G > A (maternal) + c.1787 A > G (paternal)
2	M	Birth	Deafness, developmental delay, short stature, strabismus, myoclonus, exercise intolerance, limb weakness, seizures, abnormal head imaging	8	N	Temporal lobe abnormal signals	N	NDUFA3	CI deficiency	c.173G > A (homozygous)
3	F	2 y	Developmental regression, developmental delay, headache & vomiting, decreased muscle strength, elevated blood lactate, abnormal head imaging	6	2.62	Multiple abnormal signals in periventricular areas, bilateral frontal lobes, cerebral peduncles, pons, etc.	N	NDUFAF5	CI deficiency	c.145 C > G (maternal) + c.836T > G (paternal)
4	M	Neonatal	Status epilepticus, seizures, cerebellar atrophy, G6PD deficiency, developmental delay, exercise intolerance, muscle weakness, gastrointestinal symptoms	7	U	U	N	SDHA	LS	c.1865G > A (paternal) + c.553 C > G (maternal)
5	M	Neonatal	Intrauterine growth retardation, premature birth, hypertrophic cardiomyopathy, heart failure, elevated blood lactate, abnormal head imaging, vomiting/spitting up	7	12	Head CT: Possible neonatal hypoxic-ischemic encephalopathy	N	NDUFS1	CI deficiency	c.233G > A (paternal) + c.616G > T (maternal)
6	F	9 m	Developmental delay, developmental regression, hyperlactatemia, bilateral hip dysplasia, abnormal head imaging, short stature, myoclonus, exercise intolerance	7	3	Symmetrical abnormal signals mainly in periventricular white matter & centrum semiovale, involving corpus callosum	N	NDUFS1	CI deficiency	c.64 C > T (paternal) + c.845 A > G (maternal)
7	M	13 y	Developmental delay, developmental regression, high muscle tone, exercise intolerance, facial muscle tension, scoliosis, dental disorder, hyperlactatemia, multiple abnormal signals on cranial MRI	6	U	Multiple abnormal signals	N	NDUFAF5	CI deficiency	c.165_166del (paternal) + c.434T > A (maternal)
8	M	2 y	Developmental delay, Repeated fever, heart failure, myocardial damage, hepatomegaly, elevated blood lactate, abnormal head imaging, exercise intolerance	7	6.2	Spotty abnormal signals near the ventricular body; small ischemic focus?	N	TAZ	Barth syndrome	c.758G > A (homozygous)
9	F	1 y	Short stature, recurrent hypoglycemia, trilineage cytopenia, post-liver transplantation, elevated blood lactate, abnormal head imaging	6	7.37	Left hippocampus slightly plump with locally uneven signal; MRS: Normal	N	MPV17	MPV17-related disorder	c.293 C > T (homozygous)
10	F	1 y	Hypertrophic cardiomyopathy, hyperlactatemia, subclinical hypothyroidism, exercise intolerance, gastrointestinal symptoms	5	U	U	N	ACAD9	CI deficiency	c.1594 C > T (maternal) + c.1737T > G (paternal)
11	M	5 m	Vomiting, abnormal myocardial enzymes, premature beats, myocardial hypertrophy, liver function impairment, elevated blood lactate, exercise intolerance, limb weakness	5	6.5	U	N	SLC25A26	SLC25A26 defect	c.83 A > G (maternal) + c.490T > C (paternal)
12	M	4 m	Seizures, developmental delay, elevated blood lactate, exercise intolerance, limb weakness, abnormal head imaging	7	4.39	Brain atrophy; bilateral thalamic symmetric lesions; multiple abnormal signals	Y	COQ4	LS	c.190 C > T (maternal) + c.533G > A (paternal)
13	M	1 y	developmental delay, developmental regression, exercise intolerance, limb weakness, seizures	5	U	U	N	ECHS1	LS	c.739G > A (paternal)+ c.268G > A (maternal)
14	M	1 y	Developmental regression, hypothyroidism, central diabetes insipidus, elevated blood lactate, abnormal head imaging	6	10.7	Multiple intracranial abnormal signals with cerebellar and brainstem swelling	N	NAXE	LS	c.310_314dup (maternal) + c.713T > G (paternal)
15	M	3 m	Premature birth, ARDS, seizures, growth & developmental delay, microcephaly, deafness, elevated blood lactate, abnormal head imaging	8	6.2	Multicystic encephalomalacia-like changes with atrophy; bilateral subdural effusions	N	FOXRED1	CI deficiency	c.352 C > T (maternal) + c.1054 C > T (paternal)
16	F	2 y	Seizures, developmental delay, unsteady gait, exercise intolerance; sister & brother had variants at same sites (both deceased); abnormal head imaging	6	N	Symmetrical patchy abnormal signals in the bilateral cerebellar flocculi and cerebellar hemispheres	Y	NAXE	LS	c.733 A > C (homozygous)

Barth syndrome: Barth Syndrome (TAZ Mutation); CI deficiency: Complex I Deficiency; LS: Leigh Syndrome; MPV17-related: MPV17-Related Hepatocerebral Mitochondrial Disease; SLC25A26 defect: SLC25A26 Deficienc

**Table 5:** Clinical and genetic characteristics of pediatric patients with mitochondrial DNA (mtDNA) mutations (*n* = 31)

ID	SEX	Age of onset	Clinical presentation	Score	Lactate (mmol/L)	Imaging Findings	Family history	Gene	Phenotype	Mutation and Heteroplasmy Level
1	M	16y	Sensorineural deafness, dilated cardiomyopathy, heart failure, myocardial damage, pre-excitation syndrome, hepatomegaly, short stature, abnormal cranial imaging, exercise intolerance, limb weakness, elevated blood lactate	8	4.48	Symmetric T1 hyperintensity in bilateral putamen and head of caudate nucleus; metabolic disorder primarily considered	N	MT-ND1	MELAS	m.3761 C > A, 39.26%
2	M	1y	Refractory epilepsy, developmental delay, developmental regression, exercise intolerance, muscle weakness, short stature, malnutrition, feeding intolerance, abnormal cranial imaging, elevated blood lactate	9	3	Possible subacute lacunar infarct in left thalamus; suspicious abnormal signal foci in bilateral basal ganglia; localized atrophy of frontal/temporal gyri with widened sulci	N	MT-ND1	LS	m.3700G > A, 99.5%
3	F	1y	Developmental delay, short stature, exercise intolerance, decreased muscle strength, gastrointestinal symptoms	5	U	U	N	MT-ATP6	LS	m.8989G > C, 89%
4	M	1 m	Recurrent vomiting, refractory epilepsy, developmental delay, elevated blood lactate, abnormal cranial imaging	6	3.9	Multiple abnormal signals in right temporoparietal lobe, insula, and lentiform nucleus	N	MT-ATP6	LS	m.8993T > G, 97.96%
5	M	3y	Ptosis, developmental delay, epilepsy, tracheostomy, elevated blood lactate, abnormal cranial imaging	6	2.8	Abnormal signals in bilateral thalamus, midbrain, pons, medulla oblongata, and cerebellar dentate nuclei	N	MT-ND5	LS	m.13042G > A, 88%
6	F	4 m	Liver dysfunction, hyperlactatemia, susceptibility to infectious fever, exercise intolerance, vomiting, malnutrition	5	3.1	U	N	MT-ND6	Undefined	m.14484T > C, 100%
7	M	2y	Developmental regression, abnormal cranial MRI, exercise intolerance, limb muscle weakness, vomiting	6	U	Abnormal signals in bilateral cerebral white matter, posterior limb of internal capsule, brainstem, and cerebellar dentate nuclei	N	MT-ND6	LS	m.14484T > C, 99.67%
8	M	2y	Vomiting, headache, visual impairment, Epilepsy, hypertrichosis, elevated blood lactate, abnormal cranial imaging, short stature	7	5.2	Multiple patchy abnormal signals in bilateral frontal, occipital, and right parietal lobes	N	MT-TL1	MELAS	m.3243 A > G, 88%
9	F	6y	Epilepsy, stroke-like episode, developmental delay, short stature, **family history of diabetes (multiple cases)**, elevated blood lactate level, abnormal cranial imaging	7	7	Abnormal signals in bilateral lentiform nuclei	Y	MT-TL1	MELAS	m.3243 A > G, 67.26%
10	M	10y	Epilepsy, stroke-like episodes, blurred vision, short stature, developmental regression, elevated blood lactate, abnormal cranial imaging	7	3.2	CT: symmetric calcifications in bilateral basal ganglia, low-density shadows in bilateral temporoparieto-occipital regions	Y	MT-TL1	MELAS	m.3243 A > G, 46.28%
11	M	7y	Epilepsy, short stature, deafness, elevated blood lactate, abnormal cranial imaging	6	5	Widespread abnormal signals in bilateral temporo-occipital lobes and right frontotemporal lobe; MRS: ↓NAA peak, ↑Lactate peak	N	MT-TL1	MELAS	m.3243 A > G, 96%
12	F	13y	Dilated cardiomyopathy, abdominal pain/vomiting, exercise intolerance, limb weakness, short stature	5	U	U	N	MT-TL1	MELAS	m.3243 A > G, 63.2%
13	M	2y5m	PDA closure, antral gastritis, recurrent vomiting with abdominal pain, maternal history of 3 miscarriages, epilepsy, liver damage, ventricular tachycardia, elevated blood lactate, abnormal cranial imaging, short stature	7	6	Ill-defined gray-white matter junction with abnormal signals in right parieto-occipital lobe adjacent to posterior horn of lateral ventricle	Y	MT-TL1	MELAS	m.3243 A > G, 85%
14	M	8y	Post-exercise vomiting with headache, epilepsy, stroke-like episodes, short stature, hypertrichosis, liver damage, hearing impairment, elevated blood lactate	6	5.23	No significant brain parenchymal abnormalities; slightly thickened frontoparietal meninges	N	MT-TL1	MELAS	m.3243 A > G, 50%
15	M	15y	Hypertrophic cardiomyopathy, myocardial damage, elevated blood lactate, gastrointestinal symptoms, exercise intolerance, Paternal grandmother’s sudden death	5	2.56	Normal	Y	MT-T1	MM	m.4300 A > G, 79%
16	M	6y	Refractory epilepsy, post-brain surgery, elevated blood lactate, developmental regression, abnormal cranial imaging, exercise intolerance, limb weakness	7	2.96	Post-epilepsy surgery: Postoperative changes in right occipital lobe, multiple abnormal signals in right frontoparieto-occipital lobes	N	MT-TL1	MELAS	m.3243 A > G, 88%
17	M	5y9m	Febrile convulsions in infancy, absence seizures, abnormal cranial MRI, hyperlactatemia, developmental delay (sister deceased due to convulsions, untested)	6	7.31	Suspicious white matter abnormal signal in left periventricular region	Y	MT-TL1	MELAS	m.3243 A > G, 78.28%
18	M	4y	Epilepsy, stroke-like episodes, short stature, elevated blood lactate, myoclonus, exercise intolerance, limb weakness	6	3.2	Normal	N	MT-TL1	MELAS	m.3243 A > G, 68%
19	F	11y	Developmental regression, headache, aphasia, sensorineural deafness, secondary epilepsy, short stature, elevated blood lactate, abnormal cranial imaging	7	2.59	Multiple patchy abnormal signals in bilateral front temporoparietal subcortex, basal ganglia, and cerebral peduncles; MRS: ↓NAA peak, ↑Lactate peak	N	MT-TL1	MELAS	m.3243 A > G, 78%
20	F	6y	Chronic pancreatitis, myocardial damage, hyperlactatemia, antral gastritis, liver damage, moderate malnutrition, elevated blood lactate, gastrointestinal symptoms, exercise intolerance	5	11.2	Normal	N	MT-TL1	MELAS	m.3243 A > G, 96.3%
21	M	6y	Epilepsy, generalized hypertrichosis, emaciation, abnormal cranial MRI, gastrointestinal symptoms, exercise intolerance, limb weakness	6	U	Abnormal signal in left temporal horn	N	MT-TL1	MELAS	m.3243 A > G, 50%
22	F	4y	Short stature, hypertrophic cardiomyopathy, epilepsy, post-tracheostomy, liver damage, elevated blood lactate, abnormal cranial imaging, vomiting/abdominal distension	7	2.4	Multiple ischemic foci in midbrain, pons, posterior medulla, bilateral cerebellar hemispheres, bilateral parieto-occipital lobes, thalamus, and putamen; symmetric basal ganglia calcifications; MRS: ↓AA peak, ↑Lactate peak	N	MT-TL1	MELAS	m.3243 A > G, 81.6%
23	M	3y7m	Convulsions, vomiting, headache, exercise intolerance (Maternal carrier; Maternal grandmother: deafness, diabetes; Maternal uncle: Kallmann syndrome [KAL1 gene deletion])	5	U	Normal	Y	MT-TL1	MELAS	m.3243 A > G, 58.28%
24	F	5y	Multiple febrile convulsions, epilepsy, stroke-like episodes, exercise intolerance, recurrent abdominal pain, chest wall deformity, elevated blood lactate	5	2.4	Normal	N	MT-TL1	MELAS	m.3243 A > G, 50.64%
25	M	Neonatal	Developmental delay, easy falling when walking, exercise intolerance, muscle weakness, short stature, gastrointestinal symptoms, abnormal EMG, myoclonus	6	U	U	N	MT-TF	MM	m.578T > C, 90%
26	M	7y	Epilepsy, stroke-like episodes, short stature, developmental delay, liver dysfunction, elevated blood lactate, abnormal cranial imaging	7	4.65	Abnormal signals in bilateral globus pallidus	N	MT-TS1	MELAS	m.7512T > C, 70%
27	M	12y	Chest tightness/dyspnea on exertion, dilated cardiomyopathy, myocardial damage, cardiac insufficiency, premature beats, sleep apnea, elevated blood lactate, exercise intolerance, muscle weakness	5	5.8	Normal	N	MT-TL1	MELAS	m.3243 A > G, 90%
28	M	1y	Epilepsy, developmental delay, short stature, liver dysfunction, elevated blood lactate, abnormal cranial imaging (maternal epilepsy)	8	7.37	Left medial parieto-occipital lesion; MRA normal; ↑Cho/Cr ratio, ↓NAA peak	Y	MT-TL1	MELAS	m.3243 A > G, 66.7%
29	M	1y8m	Arrhythmia, unsteady gait, positive finger-nose test, abnormal cranial MRI, developmental delay, elevated blood lactate, exercise intolerance	6	4.61	Symmetrical abnormal signals in bilateral cerebral peduncles, midbrain, pons and left external capsule	N	MT-TK	LS	m.8344 A > G, 82.21%
30	M	4y	Unsteady gait, weakness in upper limbs/neck, external ophthalmoplegia, abnormal EMG, elevated blood lactate, short stature, gastrointestinal symptoms	5	2.8	Normal	N	ND4	LS	m.11,240 C > T, 99.2%
31	M	4y	Epilepsy, hyperlactatemia, stroke-like attack, myocarditis, abnormal cranial imaging, familial diabetes, exercise intolerance	7	10.8	Multiple calcification foci around the bilateral ventricles. MRS: ↓AA peak, ↑Lactate peak.	Y	MT-TL1	MELAS	m.3243 A > G, 75.6%

LS: Leigh Syndrome; MELAS: Mitochondrial Encephalomyopathy, Lactic Acidosis, and Stroke-like Episodes; MM: Mitochondrial Myopathy

### Predominance of m.3243 A > G and its pathophysiological implications

The *MT-TL1* m.3243 A > G mutation was the most frequent mtDNA variant in our cohort, present in 60% of mtDNA-positive cases. This mutation disrupts mitochondrial protein synthesis by altering the structure and function of tRNA^Leu(UUR), leading to defective oxidative phosphorylation (OXPHOS) and a consequent reduction in ATP production [13, 14]. Clinically, m.3243 A > G is most commonly associated with MELAS but is also the primary genetic cause of maternally inherited diabetes and deafness (MIDD) [14, 15].

A notable finding in our study was that, despite a consistent maternal family history of diabetes mellitus among all probands, none had developed diabetes at the time of last follow-up. This observation reflects the incomplete penetrance and delayed onset of some systemic manifestations in m.3243 A > G carriers, which is closely linked to the typical age of MIDD onset. MIDD typically manifests in early to middle adulthood, most often between the ages of 20 and 40, although pediatric onset has been reported [16], The absence of overt diabetes in our pediatric carriers likely reflects their young age relative to this typical onset window, combined with incomplete penetrance and potentially lower heteroplasmy levels in pancreatic tissue during early life [14]. It underscores the necessity of ongoing metabolic surveillance in asymptomatic individuals and in those without current metabolic comorbidities, particularly as β-cell reserve is expected to decline with age, and cumulative metabolic stress may precipitate hyperglycemia in later years.

The pathogenesis of MIDD differs fundamentally from that of type 1 and type 2 diabetes [15]. In MIDD, mitochondrial dysfunction within pancreatic β-cells compromises ATP production, an essential signal for glucose-stimulated insulin secretion. Progressive β-cell loss due to oxidative stress and apoptosis further impairs insulin release, whereas peripheral insulin resistance plays only a minor role—contrasting sharply with type 2 diabetes, where insulin resistance predominates, and type 1 diabetes, which is driven by autoimmune β-cell destruction [16].

The disease course is generally insidious, with initially mild but progressively worsening hyperglycemia. Hearing loss is a common comorbidity, reflecting the shared susceptibility of cochlear hair cells to mitochondrial dysfunction.

From a clinical perspective, these findings highlight the need for structured metabolic monitoring in m.3243 A > G carriers, starting in late childhood or early adolescence. Recommended protocols include regular assessment of fasting plasma glucose, HbA1c, and, where feasible, oral glucose tolerance tests. Lifestyle counseling to maintain insulin sensitivity should be implemented proactively. Early identification of impaired glucose metabolism enables timely intervention, helps preserve residual β-cell function, and may prevent metabolic crises. Furthermore, understanding the mitochondrial basis of diabetes risk should guide pharmacologic decision-making, avoiding agents with potential mitochondrial toxicity and prioritizing individualized strategies aimed at preserving β-cell function.

### Diversity of nDNA mutations and early-onset phenotypes

In our cohort, nuclear DNA (nDNA) variants were detected in 12 genes, most involving complex I structure or assembly (e.g., *NDUFS1*,* NDUFAF5*,* SDHA*). These patients typically presented in infancy or early childhood, with a higher prevalence of metabolic instability—such as hypoglycemia and hyperlactatemia—consistent with international findings that nDNA-related mitochondrial disorders are often early-onset, multisystemic, and more severe [17]. Notably, *TAZ* and *MPV17* defects were more frequent in our series than in large international registries, possibly reflecting ethnic background, local referral patterns, or broader genetic screening strategies.

*TAZ*, responsible for Barth syndrome, impairs cardiolipin remodeling and destabilizes mitochondrial cristae and respiratory chain supercomplexes, leading to cardiomyopathy, skeletal myopathy, and neutropenia [18]. Our patient with homozygous *TAZ* c.758G > A (onset at 2 years) also showed periventricular brain lesions, indicating that CNS involvement can occur beyond the classic cardio–skeletal phenotype. *MPV17* deficiency, a key mitochondrial DNA maintenance disorder, predominantly manifests as early-onset hepatocerebral disease with high rates of liver failure, lactatemia, and hypoglycemia, and mortality of up to 75% in infancy [19, 20]. Our case with homozygous *MPV17* c.293 C > T (p.P98L) had hepatic, hematologic, and CNS features, improving after liver transplantation.

These observations emphasize the need to consider nDNA defects in infants with multisystem disease, particularly given the distinct phenotypic profile. While nDNA mutations are associated with significantly earlier onset and higher rates of developmental delay and abnormal brain imaging, the overall spectrum of multisystem involvement appears comparable between nDNA and mtDNA groups. Given the overlapping phenotypes between nDNA and mtDNA disorders, we recommend a concurrent diagnostic approach utilizing both whole-exome sequencing for nDNA variants and targeted mtDNA analysis to enable timely diagnosis and targeted management [10]. This parallel testing strategy optimizes diagnostic yield while minimizing delays in identifying the underlying genetic cause.

### Neuroimaging as a window into genotype-specific disease evolution

Neuroimaging findings in mitochondrial disorders demonstrate distinctive, and sometimes genotype-specific, patterns that serve as important diagnostic and prognostic clues [21]. MELAS typically presents with acute or subacute cortical and subcortical lesions that extend beyond vascular territories, showing migratory or reversible changes, often accompanied by cortical swelling and diffusion restriction. LS is characterized by symmetric involvement of the basal ganglia, thalamus, and brainstem, with early focal necrosis and progressive cystic degeneration or atrophy in later stages. POLG-related disease often shows bilateral occipital or temporal cortical involvement, sometimes with cerebellar atrophy. Kearns–Sayre syndrome frequently manifests as periventricular white matter signal abnormalities, thinning of the corpus callosum, and ventriculomegaly, whereas Leber hereditary optic neuropathy (LHON) in the acute phase may show increased signal in the optic nerves and chiasm. It should also be noted that white matter hyperintensities can occur in LHON outside of Harding syndrome, expanding the neuroimaging spectrum of this condition [16, 22, 23].Our cohort showed imaging patterns that were broadly consistent with these established features but provided unique insights through longitudinal follow-up. MELAS patients demonstrated the classical cross-vascular distribution and lesion migration, whereas *MT-ATP6*–related LS cases displayed a distinct basal ganglia–brainstem–cortical progression pattern. Notably, *NDUFS1*-related complex I deficiency evolved from focal cortical metabolic injury with cystic changes to diffuse leukoencephalopathy, a progression that has been rarely documented in such a clearly sequential manner. Literature review confirms that complex I deficiency most commonly involves the bilateral basal ganglia and brainstem, sometimes extending to the thalamus, with later development of diffuse white matter disease [21]. Magnetic resonance spectroscopy (MRS) often reveals elevated lactate peaks, reflecting localized metabolic failure [21]. The dynamic cortical-to-white matter evolution seen in our NDUFS1 cases may represent an underrecognized trajectory of complex I-related brain injury, highlighting the value of long-term imaging surveillance.

These observations underscore the importance of incorporating neuroimaging patterns into diagnostic algorithms for pediatric mitochondrial disorders, especially when genetic testing is delayed or unavailable. Early recognition of genotype-associated imaging features—such as migratory cortical lesions in MELAS, symmetric basal ganglia and brainstem lesions in LS, or progressive white matter involvement following focal cortical damage in complex I deficiency—can guide targeted genetic investigations and anticipate functional decline [16, 22, 23]. Clinically, this enables optimization of follow-up intervals, focusing imaging on structures most at risk, and initiating timely interventions while neurological function is still preserved, with the ultimate aim of delaying irreversible injury.

### High-risk prognostic triad and mortality patterns

A major novel finding of our study was the consistent identification of a “neuroimaging–metabolic–cardiac/respiratory failure” triad in all fatal cases. All six deceased patients exhibited profound neuroimaging abnormalities together with marked hyperlactatemia (≥ 6 mmol/L). Five required ventilatory support during the course of their illness, and five ultimately died from cardiac or circulatory failure, with several experiencing terminal respiratory compromise prior to death.

These observations are in line with recent large-scale international data from Ivaniuk et al. [24], which identified clinical phenotype, genotype, and age as the primary determinants of mortality risk in mitochondrial disease. In that multicenter cohort, patients with hepatocerebral syndromes, mitochondrial cardiomyopathy, and LS had the shortest survival times, whereas those with neuromuscular phenotypes such as chronic progressive external ophthalmoplegia (CPEO) demonstrated comparatively favorable outcomes. Furthermore, respiratory complications—particularly respiratory failure and pulmonary infections—were among the leading causes of death, with the highest prevalence in pediatric patients. Our findings not only corroborate these international results but also expand upon them by demonstrating that respiratory failure in our pediatric cohort frequently co-occurred with severe metabolic crisis and cardiac dysfunction, forming a reproducible triad that may signal a particularly aggressive disease trajectory. This pattern was especially prominent in patients with nDNA-related mutations, who required more intensive and prolonged respiratory support and experienced more rapid multisystem decline. The higher frequency of this triad in our series compared to international cohorts may reflect differences in genetic backgrounds, phenotype distribution, or the availability and timing of advanced respiratory interventions in pediatric care.

Recognition of this triad carries immediate clinical implications. It provides a practical framework for risk stratification, enabling early identification of patients at the greatest risk of adverse outcomes. For such high-risk individuals—particularly those with multisystem involvement and evidence of respiratory compromise—intensified multidisciplinary monitoring should be implemented, with proactive interventions targeting both cardiac and respiratory systems, along with measures to stabilize metabolic status. Tailored supportive strategies, including timely initiation of ventilatory assistance, vigilant cardiac surveillance, and prevention of respiratory infections, may help prolong survival and preserve quality of life. In parallel, early and open palliative care discussions can ensure that the intensity of treatment remains consistent with family preferences and the anticipated clinical trajectory [25].

### Implications for diagnosis and management

Our findings reinforce the importance of integrating genotype–phenotype correlations into clinical workflows. In early-onset, multisystem cases with metabolic instability, prompt nDNA testing is warranted, whereas recurrent stroke-like episodes with epilepsy in older children should raise suspicion for mtDNA mutations, particularly m.3243 A > G [8, 17]. Dynamic neuroimaging should be standardized as part of follow-up protocols, given its value in tracking disease evolution and correlating with functional decline. Finally, family-based genetic counseling should address both immediate management and long-term surveillance for potential late-onset complications such as diabetes, hearing loss, and cardiomyopathy [17].

Taken together, this study broadens the clinical and genetic landscape of pediatric MDs in China, identifies genotype-specific clinical patterns, and introduces a refined high-risk prognostic triad that incorporates both cardiac and respiratory failure. These insights offer practical guidance for individualized monitoring and therapeutic planning, while also setting the stage for future multicenter research aimed at validating prognostic markers and exploring targeted interventions.

### Limitations

Several limitations of this study should be acknowledged. First, as a single-center study, our findings may be subject to referral bias. Shanghai Children’s Medical Center serves as a tertiary referral center for complex pediatric cases, which may lead to an overrepresentation of severe phenotypes and specific mutation types compared to the general population. This referral bias could influence the observed mutation frequencies and phenotype distributions, limiting the generalizability of our results to broader clinical settings. Second, our study’s statistical power was limited by the relatively small sample size, particularly in subgroup analyses. Several clinically relevant comparisons, such as stroke-like episodes between mtDNA and nDNA groups (*P* = 0.081) and abnormal brain imaging (*P* = 0.062), approached but did not reach statistical significance. These trends may represent true biological differences that would become statistically significant with larger sample sizes. Future collaborative studies with larger cohorts are warranted to confirm these preliminary observations. Third, heteroplasmy levels were measured only in peripheral blood. Mitochondrial DNA heteroplasmy can vary significantly across different tissues, and blood heteroplasmy levels may not accurately reflect the mutation burden in clinically affected organs such as brain, muscle, or liver. Multi-tissue heteroplasmy analysis, including muscle biopsy when clinically indicated, would provide more accurate genotype-phenotype correlations and better predict clinical severity. Future studies incorporating tissue-specific heteroplasmy measurements could enhance our understanding of the relationship between mutation load and clinical manifestations. Fourth, neuroimaging protocols varied over the 10-year study period, with earlier examinations utilizing standard MRI sequences while later studies incorporated advanced techniques such as diffusion-weighted imaging and magnetic resonance spectroscopy. This temporal heterogeneity in imaging methodologies may have introduced variability in lesion detection sensitivity and influenced the interpretation of neuroimaging findings. Standardization of imaging protocols would strengthen future comparative analyses. Finally, as the study was observational in nature, treatment strategies were not uniform, limiting our ability to assess therapeutic efficacy or disease-modifying effects. The identification of specific genotype-phenotype correlations and prognostic indicators offers opportunities for targeted clinical trial design and personalized treatment approaches. Future investigations should explore how these findings might inform patient selection criteria, endpoint selection, and therapeutic development in mitochondrial medicine.

Future research should aim to validate these findings in multicenter, prospective studies with larger sample sizes, standardized neuroimaging protocols, and multi-tissue heteroplasmy assessments. Integration of functional assays and longitudinal clinical outcome measures will be critical for translating genotype–phenotype correlations into targeted interventions.

## Conclusion

In conclusion, this study characterizes the genetic and phenotypic diversity of pediatric MDs in China, with m.3243 A > G as the most common mtDNA mutation and a predominance of complex I-related defects among nDNA cases. Genotype-driven clinical patterns, particularly the earlier onset and metabolic instability in nDNA mutations versus the higher epilepsy and stroke-like episode rates in mtDNA cases, provide valuable guidance for diagnostic prioritization. Dynamic neuroimaging proved essential in revealing disease-specific progression patterns, while the identification of a “neuroimaging–metabolic–cardiac/respiratory failure” triad as a marker of high mortality risk offers a practical tool for prognostic assessment. These insights not only enhance our understanding of MD in the Chinese pediatric population but also inform tailored diagnostic, monitoring, and therapeutic strategies.

## Data Availability

All the data during this study are included in this published article.

## Abbreviations

ACMG: American College of Medical Genetics and Genomics
ATP: Adenosine triphosphate
CI: Complex I (NADH: ubiquinone oxidoreductase)
CNS: Central nervous system
DNA: Deoxyribonucleic acid
HGVS: Human Genome Variation Society
MDC: Mitochondrial Disease Criteria
MD: Mitochondrial disorder
MELAS: Mitochondrial encephalomyopathy, lactic acidosis, and stroke–like episodes
LS: Leigh Syndrome
MRS: Magnetic resonance spectroscopy
mtDNA: Mitochondrial DNA
NGS: Next–generation sequencing
nDNA: Nuclear DNA
NARP: Neuropathy, ataxia, and retinitis pigmentosa
OXPHOS: Oxidative phosphorylation
SD: Standard deviation
SEEG: Stereo–electroencephalography
WES: Whole–exome sequencing

## References

[1] Wen H, Deng H, Li B, Chen J, Zhu J, Zhang X, et al. Mitochondrial diseases: from molecular mechanisms to therapeutic advances. Signal Transduct Target Ther. 2025;10(1):9. 39788934 10.1038/s41392-024-02044-3 PMC11724432

[2] Fernandez-Vizarra E, Zeviani M. Mitochondrial disorders of the OXPHOS system. FEBS Lett. 2021;595(8):1062–106. 33159691 10.1002/1873-3468.13995

[3] Liu BH, Xu CZ, Liu Y, Lu ZL, Fu TL, Li GR, et al. Mitochondrial quality control in human health and disease. Mil Med Res. 2024;11(1):32. 38812059 10.1186/s40779-024-00536-5 PMC11134732

[4] Wong TS, Belaramani KM, Chan CK, Chan WK, Chan WL, Chang SK, et al. Mitochondrial diseases in Hong kong: prevalence, clinical characteristics and genetic landscape. Orphanet J Rare Dis. 2023;18(1):43. 36859275 10.1186/s13023-023-02632-6 PMC9979401

[5] Feeney CL, Lim AZ, Fagan E, Blain A, Bright A, Maddison J, et al. A case-comparison study of pregnant women with mitochondrial disease - what to expect? BJOG. 2019;126(11):1380–9. 30801962 10.1111/1471-0528.15667 PMC6767368

[6] Thomas RH, Hunter A, Butterworth L, Feeney C, Graves TD, Holmes S, et al. Research priorities for mitochondrial disorders: current landscape and patient and professional views. J Inherit Metab Dis. 2022;45(4):796–803. 35543492 10.1002/jimd.12521 PMC9429991

[7] Hurko O. Drug development for rare mitochondrial disorders. Neurotherapeutics. 2013;10(2):286–306. 23430661 10.1007/s13311-013-0179-4 PMC3625383

[8] Stenton SL, Prokisch H. Genetics of mitochondrial diseases: identifying mutations to help diagnosis. EBioMedicine. 2020;56:102784. 32454403 10.1016/j.ebiom.2020.102784 PMC7248429

[9] Liu Z, Pan K, Wang M, Jin Y, Yang W, Chen K, et al. Novel pathogenic MtDNA variants in Chinese children with neurological mitochondrial disorders. Ann Clin Transl Neurol. 2025;12(3):586–601. 39913609 10.1002/acn3.52315 PMC11920736

[10] Tsang MHY, Kwong AKY, Chan KLS, Fung JLF, Yu MHC, Mak CCY, et al. Delineation of molecular findings by whole-exome sequencing for suspected cases of paediatric-onset mitochondrial diseases in the Southern Chinese population. Hum Genomics. 2020;14(1):28. 32907636 10.1186/s40246-020-00278-0 PMC7488033

[11] Shayota BJ. Biomarkers of mitochondrial disorders. Neurotherapeutics. 2024;21(1):e00325. 38295557 10.1016/j.neurot.2024.e00325 PMC10903091

[12] Morava E, van den Heuvel L, Hol F, de Vries MC, Hogeveen M, Rodenburg RJ, et al. Mitochondrial disease criteria: diagnostic applications in children. Neurology. 2006;67(10):1823–6. 17130416 10.1212/01.wnl.0000244435.27645.54

[13] Ryytty S, Hämäläinen RH. The mitochondrial m.3243A > G mutation on the dish, lessons from in vitro models. Int J Mol Sci. 2023;24(17). 10.3390/ijms241713478 PMC10487608 37686280

[14] Robinson KN, Terrazas S, Giordano-Mooga S, Xavier NA, THE ROLE OF HETEROPLASMY IN THE DIAGNOSIS AND MANAGEMENT OF MATERNALLY INHERITED DIABETES AND DEAFNESS. Endocr Pract. 2020;26(2):241–6. 31682520 10.4158/EP-2019-0270

[15] Chanoine JP, Thompson DM, Lehman A. Diabetes associated with maternally inherited diabetes and deafness (MIDD): from pathogenic variant to phenotype. Diabetes. 2025;74(2):153–63. 39556456 10.2337/db24-0515 PMC11755681

[16] Kandemirli SG, Al-Dasuqi K, Aslan B, Goldstein A, Alves C. Overview of neuroimaging in primary mitochondrial disorders. Pediatr Radiol. 2025;55(4):765–91. 39937244 10.1007/s00247-025-06172-y

[17] Gorman GS, Chinnery PF, DiMauro S, Hirano M, Koga Y, McFarland R, et al. Mitochondrial diseases. Nat Rev Dis Primers. 2016;2:16080. 27775730 10.1038/nrdp.2016.80

[18] El-Hattab AW, Wang J, Dai H, Almannai M, Staufner C, Alfadhel M, et al. MPV17-related mitochondrial DNA maintenance defect: new cases and review of clinical, biochemical, and molecular aspects. Hum Mutat. 2018;39(4):461–70. 29282788 10.1002/humu.23387

[19] Ghosh S, Iadarola DM, Ball WB, Gohil VM. Mitochondrial dysfunctions in Barth syndrome. IUBMB Life. 2019;71(7):791–801. 30746873 10.1002/iub.2018 PMC6586490

[20] El-Hattab AW, Li FY, Schmitt E, Zhang S, Craigen WJ, Wong LJ. MPV17-associated hepatocerebral mitochondrial DNA depletion syndrome: new patients and novel mutations. Mol Genet Metab. 2010;99(3):300–8. 20074988 10.1016/j.ymgme.2009.10.003

[21] Alves C, Whitehead MT. Advancing the neuroimaging diagnosis and Understanding of mitochondrial disorders. Neurotherapeutics. 2024;21(1):e00324. 38306952 10.1016/j.neurot.2024.e00324 PMC10903090

[22] Saneto RP, Friedman SD, Shaw DW. Neuroimaging of mitochondrial disease. Mitochondrion. 2008;8(5–6):396–413. 18590986 10.1016/j.mito.2008.05.003 PMC2600593

[23] Gropman AL. Neuroimaging in mitochondrial disorders. Neurotherapeutics. 2013;10(2):273–85. 23208728 10.1007/s13311-012-0161-6 PMC3625392

[24] Ivaniuk A, Anselm IA, Bowen A, Cohen BH, Eminoglu FT, Estrella J, et al. Characterization of factors associated with death in deceased patients with mitochondrial disorders: A multicenter Cross-Sectional survey. Neurology. 2025;104(4):e209779. 39883904 10.1212/WNL.0000000000209779 PMC11781783

[25] Parikh S, Goldstein A, Karaa A, Koenig MK, Anselm I, Brunel-Guitton C et al. Patient care standards for primary mitochondrial disease: a consensus statement from the mitochondrial medicine society. Genet Med. 2017;19(12). 10.1038/gim.2017.107 PMC7804217 28749475

